# Evaluation of a custom-made mandibular repositioning device for the treatment of obstructive sleep apnoea syndrome

**DOI:** 10.1038/s41415-023-6267-x

**Published:** 2023-09-22

**Authors:** Lampros Flouris, Brian Millar

**Affiliations:** 41415361107001 Grange Green Dental Practice, 42 Grange Road, Billericay, Essex, CM11 2RG, UK; 41415361107002 Faculty of Dentistry, Oral and Craniofacial Sciences, King´s College London, London, UK

## Abstract

Obstructive sleep apnoea (OSA) is a common, chronic condition that affects breathing during sleep. The gold standard for treatment is continuous positive airway pressure (CPAP) which is often not well-tolerated. Mandibular repositioning appliances (MRDs) are an alternative that dentists may be requested to provide.

The purpose of this audit is to evaluate the effectiveness of an MRD in improving the Oxygen Desaturation Index (ODI) and Epworth Sleepiness Scale (ESS). A total of 52 patients diagnosed with OSA in an NHS hospital respiratory clinic were fitted with an MRD. Analysis of the digitally recorded oximeter signals was recorded among other treatment outcomes before and 6-8 weeks after the fit of the appliance.

The meta-analysis of the audit data showed supportive evidence for MRD treatment in OSA patients. There was a statistically significant reduction in ODI and ESS. The audit population consisted of 138 patients (91 men, 47 women; mean age: 49.49 ± 11.93 years). The ODI outcomes improved significantly, from 10.68 to 6.58 (p <0.02). The ESS improved significantly from 9.46 to 6.02 (p <0.01).

This audit demonstrates that MRDs are effective and should be considered as an alternative to CPAP in some specific OSA phenotypes.

## Key points

- Obstructive sleep apnoea is a common and serious medical condition that in many cases can be managed by the use of a mandibular repositioning appliance.
- Suitable appliances can easily be provided by dentists.
- The simple elastomeric appliance is effective in reducing sleep apnoea and daytime sleepiness.

## Introduction

According to the American Academy of Sleep Medicine (AASM), obstructive sleep apnoea (OSA)/hypopnoea syndrome can be defined as a state characterised by episodes of complete or partial upper airway obstruction during sleep and is usually associated with snoring, intermittent hypoxaemia and arousal from sleep.^1^

The increasing prevalence of OSA is becoming a significant problem for healthcare systems around the globe, affecting 1.8 million people in the UK. The severity of OSA is determined by the Apnea Hypopnea Index (AHI): <5/hour = normal (for adults); 5-14.9/hour = mild OSA; 15-29.9/hour = moderate OSA; and ≥30/hour = severe OSA. The available data illustrate that one in four adults have OSA (AHI ≥5/hour), and approximately one in ten has moderate to severe disease (AHI ≥15/hour).^2^

Among the frequently observed symptoms are: daytime sleepiness; increased risk for vehicle accidents and occupational accidents; cognitive impairment; significant use of medication; unemployment; an independent risk factor for hypertension; and associated with cardiovascular and cerebrovascular morbidity. OSA poses a considerable problem for public health and the economy.^3^^,^^4^

OSA contributes to the development and progression of cardiovascular conditions, such as systemic arterial hypertension, heart failure, metabolic syndrome, cardiac arrhythmia and coronary artery disease.^5^^,^^6^

The primary approach for treating patients with moderate to severe OSA and an option of treatment for patients with mild OSA is continuous positive airway pressure (CPAP).^7^^,^^8^ The primary function of CPAP is the stabilisation of the upper airway and preventing its periodic collapse during sleep.^9^

An alternative treatment for patients with low compliance/tolerance to CPAP is a mandibular repositioning device (MRD). MRDs are a well-tolerated treatment for patients suffering from OSA and those who cannot tolerate or want to avoid using CPAP.^10^^,^^11^

The AASM and the American Academy of Dental Sleep Medicine recommend the use of a custom and titratable oral appliance, rather than no treatment, for patients with mild to moderate OSA; patients with a preference for using MRDs; and patients unable to tolerate CPAP therapy or who request treatment for primary snoring (without OSA).^1^

It is recognised that both treatment modalities can reduce the AHI, improve the symptoms of sleepiness and doziness, and improve the quality of life of patients suffering from moderate to severe OSA.^12^ The primary factor affecting CPAP effectiveness is the lower tolerance. As a result, the lower adoption rate reduces the efficacy of treatment with CPAP, as was shown by Sutherland *et al.*^13^

The primary outcomes of interest are OSA level, Epworth Sleepiness Scale (ESS) (Table 1), body mass index (BMI) and Oxygen Desaturation Index (ODI). Desaturation episodes are defined as a decrease in the mean oxygen of ≥4% (over the past two minutes) that lasts for at least ten seconds. Chung *et al.*, in their study involving 475 patients at the University of Toronto, concluded that ODI> is a good predictor for AHI >5 with an accuracy of 87%, an ODI >15 for AHI >15 with an accuracy of 84%, and an ODI >30 for AHI >30 with an accuracy of 93.7%.^14^

**Table 1:** Baseline characteristics of the population studied: age, sex (men), BMI, OSA level, ESS, ODI

Characteristics of the population	Pre fit (mean ± SD)	Post fit
Age (years)	49.49 ± 11.93	52.63 ± 12.6
Sex - men	N = 91 (65.9%) Total cases: 138	N = 36 (69.2%) Total cases: 52
OSA level	Mild: n = 38 Moderate: n = 76 Severe: n = 24	Mild: n = 31 Moderate: n = 17 Severe: n = 4
ODI - 4% ODI/hr	10.68 ± 12.55	6.58 ± 6.05
BMI (kg/m2)	31.13 = -7.05	31.15 ± 7.49
ESS	9.46 ± 5.28	6.02 ± 5.53
SpO2 mean	79.35 ± 9.52	82.74 ± 7.55
Key: Plus-minus values are means ± standard deviations		

The present audit is aligned with the existing knowledge base on the efficacy of MRD therapy as an alternative to CPAP treatment. The improvement in the ODI and ESS scoring, in combination with the available literature data, allowed us to demonstrate that MRD treatment has an important role not only as first-line treatment in mild to moderate OSA, but also as a lifesaving alternative in patients unable to cope or declining CPAP.^15^^,^^16^

The definition of hypopnea is controversial since the prevalence of OSA in various populations depends on which definition is chosen. An oxygen desaturation threshold of ≥4% was chosen as this is the most frequently used.^17^^,^^18^

This audit aimed to evaluate the outcomes of a simple elastomeric MRD provided in an NHS hospital clinic for the treatment of OSA and compare it with the results of published data.

## Materials and methods

Patients were assessed in a sleep clinic at the Department of Respiratory Medicine at King's College Hospital (KCH). If patients were considered suitable for MRD therapy based on the ODI score and mild/moderate OSA and satisfaction of the inclusion criteria, they were referred to the Unit of Restorative Dentistry at KCH for a dental assessment by a specialist prosthodontist and provision of a mandibular advancement appliance where suitable. Routine dental care was carried out where necessary before the MRD could be provided.

The inclusion criteria for provision of an MRD were:

- Patients aged over 18 years with newly diagnosed low to medium level OSA
- Two or more symptoms of OSA (snoring, fragmented sleep, witnessed apnoea's, or daytime sleepiness)
- Severe cases of OSA who could not tolerate CPAP
- Satisfactory oral health and an adequate number of teeth to provide stability for the appliance
- No temporomandibular joint pain symptoms.

**Fig. 1:**
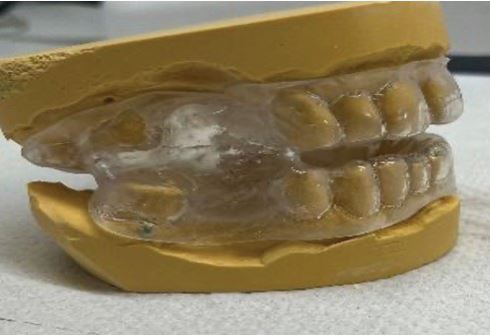
An example of the elastomeric appliance evaluated in the audit

Upper and lower alginate impressions were recorded, along with a silicone protrusive intra-occlusal record taken at 60% protrusion of the mandible, usually around 5-6 mm. The MRD was fabricated in-house from a 2 mm vacuum-formed polymer using articulated casts to create an elastomeric monoblock design (Fig. 1).

A specialist prosthodontist fitted the MRD and both verbal and written instructions were provided. The sleep clinic was informed and a review appointment was arranged for 6-8 weeks. At this appointment, the follow-up data were collected and logged in. The data were then processed and analysed, ensuring complete anonymity was secured.

This was a retrospective audit of hospital records. Data collected were anonymised. NHS ethics approval was checked and deemed unnecessary.

### Definitions of respiratory variables used

- Apnoea: the complete stop (>90%) of the nasal-oral airflow for a minimum of ten seconds. The apnoea was then classified as obstructive if it was accompanied by thoracic and abdominal effort, central if this effort was absent and mixed if both situations occurred in one single apnoea
- Hypopnea: a drop in the respiratory signal between 30% and 90%, accompanied by a decline in oxygen saturation ≥4 and/or arousal.

### Outcomes evaluated

#### Oxygen Desaturation Index and SpO2

OSA subjective symptoms seem to be correlated to oxygen desaturations. Hypoxia during apnoea periods of OSA is significant; therefore, ODI is as valuable as AHI in diagnosing and grading OSA.^19^ The oxygen monitor used was the Viatom Checkme O2 and data was downloaded with Stowood Visi scientific oximetry software.

One of the significant issues related to AHI is that features of the abnormally respiratory events are not considered. AHI is a quantitative parameter. It has been observed that in cases where the depth and duration of the apnoea attacks increase, AHI may fall.

Oxygen desaturation poses one of the most significant factors for complications related to OSA. Reflecting on the importance of oxygen desaturation events in the pathophysiology of OSA, we compared the ODI before and after the fit of an MRD. ODI calculates the number of desaturation events per hour which drop 4% below baseline levels.^20^

Raw SpO2 (oxygen saturation) data were also analysed, providing detailed information regarding OSA-related pathophysiology and are summarised in the ODI scoring.^21^

The effectiveness of MRD treatment depends on patient acceptance; therefore, compliance, side effects and withdrawal from treatment were also recorded.

#### Epworth Sleepiness Scale

The use of the ESS, a self-administrated questionnaire of eight questions, provides a measure of the subject's general level of daytime sleepiness or dozing. The ESS scores can help to classify normal subjects from patients suffering from OSA syndrome, idiopathic hypersomnia and narcolepsy. It's a measure of the probability of falling asleep in various situations.^22^

#### Body mass index

Excessive body mass increases the probability of breathing related to sleep disorders and significantly affects the range and success of the treatment.^23^ BMI records of 40+ kg/m2 were 27.39 times (95% CI: 24.64-30.46) more likely to have OSA (p <0.0001).^24^

#### OSA level

Polysomnography is the gold standard for diagnosing OSA and other sleep-related sleeping disorders. The severity of OSA is graded as:

- Mild OSA: AHI >5 events per hour
- Moderate OSA: 15 events per hour
- Severe OSA: 30 events per hour.

As reported above, the use of ODI was used for the classification of the OSA level.

## Results

### Baseline characteristics of the patients

In the review of the available audit data, 138 patients fulfilled the inclusion criteria and were provided with an MRD, including 91 men (65.9%) and 47 women (34.1%), with a mean age of 49.49 ± 11.93 years. Baseline characteristics are shown in Table 1 and showed that 8 patients (5.7%) had severe OSA and 130 patients (94.2%) had mild to moderate OSA (Table 2).

**Table 2:** Range of grading according to ESS and ODI

		Pre treatment	Post treatment
ESS	Normal (≤8)	60	39
	Mild moderate (9-14)	53	12
	Severe (15-24)	25	1
ODI	Common snoring (<5)	38	14
	Mild (5-14)	76	31
	Moderate (15-19)	16	4
	Severe (≥30)	8	3

### Follow-up

The duration of the follow-up period was 6-8 weeks and 52 returned for a follow-up appointment.

### Statistical analysis

A statistical software package (SPSS Inc, Chicago, IL) was used for data processing and statistical analysis. Continuous variables were expressed as the mean ± standard deviation and qualitative variables were expressed as a percentage.

### Treatment outcome

Following the fit of the MRD at the two-month follow-up period, the mean BMI (31.15 ± 7.49), ODI (6.58 ± 6.05) and ESS (6.02 ± 5.53) were recorded. Sleep and respiratory scores were obtained in line with the 2009 guidelines from the AASM. A decrease of at least 4% in oxygen saturation value during sleep was calculated as desaturation and the mean desaturation value in an hour was represented by ODI.

**Fig. 2:**
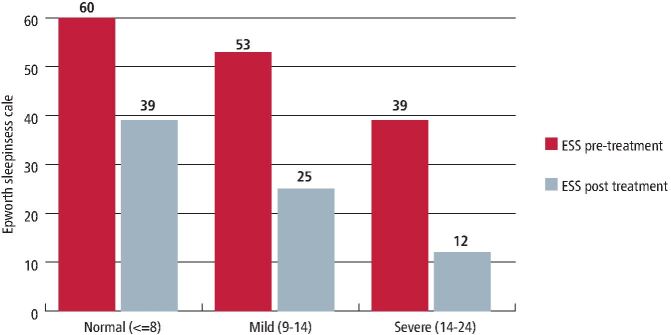
Initial ESS scores and post-MRD fit scores for each class of OSA case (red = pre MRD; grey = post MRD)

Comparison of initial and post-fit values for ODI showed a significant improvement, from 10.68 to 7.52 (p <0.02). A significance value (p-value) and 95% confidence interval of the difference are reported (Table 3). Figure 2 shows the improvement in ODI for each category of OSA.

**Table 3:** ODI outcome pre and post MRD treatment

Treatment - MRD, ODI	Result
Pre treatment	10.68 ± 12.55
Post treatment	6.58 ± 6.05

**Fig. 3:**
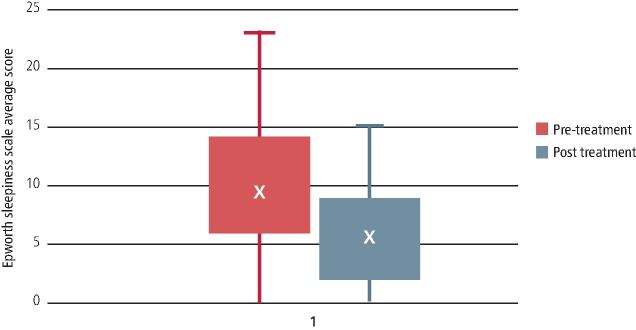
ESS values (red = pre MRD; grey = post MRD)

The outcome with respect to the ESS showed a significant improvement (Table 4) from an initial 9.46 to post-fit 6.02 (p <0.01). Figure 3 shows the improvement in ODI for each category of OSA.

**Table 4:** ESS scores pre and post MRD treatment

Treatment - MRD	Questionnaire	Range	Result
Pre treatment	ESS	0-24	9.46 ± 5.28
Post treatment	ESS	0-24	6.02 ± 5.53

The mean minimum blood oxygen saturation level during sleep improved significantly from 79.35% to 82.74% (p <0.02) as seen in Table 5.

**Table 5:** SpO2 minimum blood oxygen saturation level outcome

Treatment - MRD SpO2 min	Result
Pre treatment	79.35 ± 9.52
Post treatment	82.74 ± 7.55

BMI did not differ pre treatment and post treatment with an MRD. There was no significant improvement as seen in Table 6.

**Table 6:** BMI scores pre and post MRD

Body mass index (kg/m2)	Result
Pre treatment	31.13 ± 7.05
Post treatment	31.25 ± 7.49

### Adverse effects

In the initial period of oral appliance therapy, patients reported some mild and temporary adverse effects. Excessive salivation, dry mouth, temporomandibular joint pain and gum irritation were some of the reported issues.

## Discussion

Treatment success observed with this audit was at the upper end of the range reported in previous MRD devices in OSA. In most of the available studies, a more significant improvement in AHI, SpO2 and pharyngeal collapsibility was noted with greater protrusion.^25^^,^^26^

The results of this audit show that a custom-made appliance can be an effective and well-tolerated treatment option for the type of patients included in this audit with mild to moderate OSA and those who refuse or cannot tolerate CPAP.

This retrospective audit showed MRD therapy improving ODI. As discussed, ODI strongly correlates with the parameters used to measure sleep breathing disorders from polysomnography (PSG). In a large prospective cohort study, Chung *et al.* found that ODI had a very high accuracy in predicting AHI at cut-offs of 5, 15 and 30, with the area under receiver operating characteristic of 0.908 to 0.958. ODI >5 was a good predictor for AHI> with an accuracy of 87%, ODI >15 for AHI >15 with an accuracy of 84%, and ODI >30 for AHI >30 with an accuracy of 93.7%.^14^

Various studies performed during wakefulness have concluded that the effect of MRDs is due to the enlargement of the pharyngeal airway, remarkably in the lateral dimension.^27^ Various studies tried to link the level of airway narrowing or collapsibility as a predictive factor for effective treatment with MRD. The results are not encouraging, so other predictors should be examined.^28^

The success rate of long-term MRD therapy has been the subject of numerous studies. Eriksson *et al.*, in their ten-year follow-up study, reported that seven in ten patients in the MRD group were treated successfully in terms of objective reduction of AHI. An interesting finding in this study is the perception of a successful outcome from patients. Almost nine in ten patients considered themselves cured.

Various studies confirm that prolonged treatment with oral appliances and CPAP may result in dental changes. A systematic review done by Bertolucci and team explored the dental and skeletal changes that occurred in 18 studies in a period that varied from 2-11 years. This review concluded that the dental side effects are progressive and strongly correlated with the duration of treatment.^29^

The common belief is that the benefit of treatment for OSA outweighs the possible risk and it can be said that MRD treatment is well tolerated.

In the current audit, BMI did not change over the period of two months. Increased BMI is considered probably the most crucial risk factor for OSA.^2^ Various cross-section studies have linked increased body weight and the risk of OSA. In the same way, weight loss in OSA led to a significant decrease in apnoea frequency.^30^ The exact mechanism is not fully determined, but some studies are trying to investigate the role of leptin. Leptin, which is found to increase in obese patients, has a profound impact on the regulation of chemoreflex function and, as a result, breathing control.^31^ This poses an exciting field for research to determine if leptin levels can be a factor that should be investigated when OSA risk level is determined.

As discussed above, one of the indicators for a successful outcome is younger age. It is assumed that ageing is associated with a reduction in the upper airway dilator muscle performance and a decrease in the genioglossus negative pressure index.^32^ Considering other factors with increased prevalence within older populations, like arthritis, diabetes and cardiovascular diseases, makes successful management of OSA more challenging.^33^

Currently, there is not an ubiquitous accord upon definition of a successful treatment outcome with MRDs for OSA. Some studies in the bibliography define treatment success as the reduction in AHI below 5, 10 or even 20 events/hour of sleep. In this audit, treatment success was described as a statistically significant improvement in the means of ODI and ESS. As is apparent, success is not defined as the reduction of AHI only, as an improvement in the ESS was also recorded.

More well-designed clinical studies evaluating MRDs as an alternative therapeutic approach compared to CPAP, concerning the heterogeneity of the OSA and with more extended follow-up periods, are encouraged, to get a more definite conclusion of the therapeutic advantage and will allow the identifications of clinical features of the patients that will benefit the most from MRDs as a treatment modality. Furthermore, more studies associating the ODI with AHI are necessary, especially from an economical and practical aspect, since acquiring data from an oximeter in a regular clinic is much easier and more cost-effective than using PSG.

OSA poses a promising field for research with direct and applicable results that will positively impact the quality of life of patients and allow better treatment planning and reduction of cost to the health boards. A field of great interest is the use of mandibular advancement appliances for OSA children. For children with dental/skeletal Class II malocclusion especially, long-term use of a mandibular advancement appliance can potentially affect the mandible length and as a positive outcome, increase the posterior airway.^34^ On the other hand, patients with a skeletal and dental Class I relationship should restrict the use of a mandibular advancement appliance to only during the night, and close monitoring by an orthodontist is advised.

## Conclusion

This design of MRDs effectively reduces ODI and ESS, and there is sufficient evidence to support their use as an alternative to CPAP in specific OSA phenotypes. Based on the available evidence, sleep physicians should consider using MRDs, rather than no treatment, for adult patients with mild to moderate OSA, as well as those who cannot tolerate CPAP treatment. This recommendation aligns with the published guidance from the AASM.

Better management of OSA has a positive impact on life and probably reduces the need for more long-term care. The general consensus is that the benefits outweigh the risk if a qualified dentist provides the treatment with the appropriate knowledge, technical skills, and judgement to assess the benefit versus the risk for everyone.
